# Mutations of c-Cbl in myeloid malignancies

**DOI:** 10.18632/oncotarget.3986

**Published:** 2015-05-04

**Authors:** Shulamit Katzav, M. Lienhard Schmitz

**Affiliations:** ^1^ Developmental Biology and Cancer Research, IMRIC, Faculty of Medicine, The Hebrew University, Jerusalem, Israel; ^2^ Institute of Biochemistry, University of Giessen, Friedrichstrasse, Giessen, Germany

**Keywords:** cbl, myeloid malignancies

## Abstract

Next generation sequencing has shown the frequent occurrence of point mutations in the ubiquitin E3 ligase c-Cbl in myeloid malignancies. Mouse models revealed a causal contribution of c-Cbl for the onset of such neoplasms. The point mutations typically cluster in the linker region and RING finger domain and affect both alleles by acquired uniparental disomy. The fast progress in the detection of c-Cbl mutations is contrasted by our scarce knowledge on their functional consequences. The c-Cbl protein displays several enzymatic functions by promoting the attachment of differentially composed ubiquitin chains and of the ubiquitin-like protein NEDD8 to its target proteins. In addition, c-Cbl functions as an adapter protein and undergoes phosphorylation-dependent inducible conformation changes. Studies on the impact of c-Cbl mutations on its functions as a dynamic and versatile adapter protein, its interactomes and on its various enzymatic activities are now important to allow the identification of druggable targets within the c-Cbl signaling network.

## INTRODUCTION

Myeloid malignancies comprise three broad categories: acute myelogenous leukemia (AML) characterized by accumulation of immature myeloid cells in the bone marrow, myelodypastic syndromes (MDS) associated with ineffective hematopoiesis and chronic myeloproliferative disorders (MPDs), usually associated with an increased production of terminally differentiated myeloid cells. Despite this heterogeneity, all myeloid malignancies originate from progenitors that normally give rise to terminally differentiated cells of the myeloid series. Genetic and epigenetic events occurring early in hematopoietic stem cell maturation can lead to myeloid malignancies. Mutations can disrupt the normal course of hematopoiesis, resulting in a wide range of defects affecting the myeloid lineages [1].

Normal hematopoiesis involves a strict hierarchy of hematopoietic progenitor cells. The single primary source for the entire mammalian blood system is the hematopoietic stem cell (HSC), which is both multipotent and self-renewing in the absence of differentiation [2]. HSCs initially give rise to multipotent progenitors (MPPs), which have lost the ability to self-renew, but still maintain full-lineage differentiation potential [3]. MPPs can differentiate into common myeloid progenitors (CMPs), leading eventually to the generation of the myeloid lineage [2, 4-8].

Some myeloid malignancies are characterized by prominent and characteristic disease-causing mutations. For example, the vast majority of Polycythemia vera patients carry gain-of function mutations in Janus kinase 2 (JAK2 V617F) [9], while a hallmark of chronic myeloid leukemia (CML) is the generation of BCR-ABL fusion proteins [10]. On the other hand, two thirds of patients with myelodysplastic/myeloproliferative neoplasms (MDS/MPN) have a normal karyotype, but have several oncogenic mutations in a variety of genes encoding proteins involved in RNA splicing, chromatin modification, transcription or signaling [11]. A substantial fraction of MDS/MPN and their subtypes is characterized by the frequent occurrence of mutations in the gene encoding c-Cbl (Casitas B-lineage Lymphoma), a protein whose various enzymatic and non-enzymatic functions are now beginning to emerge.

### Architecture and function of c-Cbl proteins

**Figure 1:**
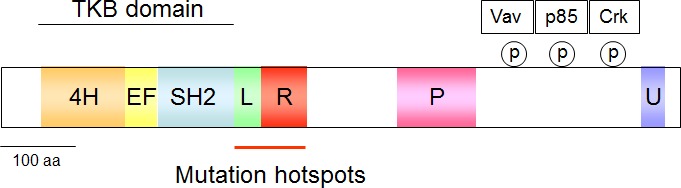
Schematic display of the c-Cbl structure The various domains are given in colors, 4H: four helical bundle, EF: EF-hand calcium-binding domain, SH2: Src homology 2, L: linker helical region, P: proline-rich, U: Ubiquitin-binding domain. The phosphorylated tyrosines in the C-terminus allow docking of the indicated proteins and the frequently mutated region is highlighted.

The attachment of ubiquitin to its client proteins regulates many different biological processes and occurs by a complex enzymatic cascade [12]. This process is critically dependent on ubiquitin activation by the E1 enzymes, followed by transfer of the activated ubiquitin to an ubiquitin-conjugating enzyme (E2) and finally, conjugation of ubiquitin to the target protein by an ubiquitin-protein ligase (E3). The E3 family of ubiquitin-protein ligases has a crucial role in substrate recognition and is of great importance in the (patho)physiology of intracellular signaling networks [13, 14]. Among E3 ligases, the small family of Cbl ubiquitin E3 ligases consists of the ubiquitously expressed proteins c-Cbl and Cbl-b as well as Cbl-c, which occur primarily in epithelial cells. All Cbl proteins share an N-terminal tyrosine-kinase-binding (TKB) domain that is followed by a RING (really interesting new gene) finger and a proline-rich domain [15]. The TKB domain is a modified SH2 (Src homology 2) domain that allows binding to phosphorylated tyrosines typically found in Cbl-interacting proteins. Cbl proteins are activated by phosphorylation at Y371 located in a linker region between the RING finger and the TKB domain [16]. This phosphorylation releases Cbl from its autoinhibited structure by triggering a conformational change that results in increased binding of the ubiquitin conjugating protein (or E2) to the RING finger of Cbl. This in turn leads to an enhanced transfer of ubiquitin from the E2 enzyme to the substrate proteins [17, 18]. The C-termini of Cbl-b and c-Cbl harbor extended proline-rich sequences and also a domain involved in ubiquitin binding and dimerization [19]. These domains allow for regulated protein/protein interactions via inducible association with SH2 and SH3 (Src homology 3) domain-containing proteins, as schematically displayed in Figure 1.

Cbl proteins can exert multiple enzymatic functions. They can attach K48-branched polyubiquitin chains, resulting in proteasomal degradation of the decorated client proteins [20]. Cbl E3 ligases can also mediate the attachment of either K63-conjugated ubiquitin chains or single ubiquitin molecules to their substrate proteins to mediate non-proteolytic regulatory functions [21]. Recent evidence shows that c-Cbl also has the ability to mediate attachment of the ubiquitin-like protein NEDD8 (neural precursor cell expressed, developmentally down-regulated 8) to substrate proteins. It was shown that c-Cbl-mediated neddylation of the TGF-β type II receptor antagonizes its ubiquitination and degradation [22]. Currently, it is not clear what molecular events guide Cbl proteins to distinguish between these four distinct enzymatic functions.

**Figure 2:**
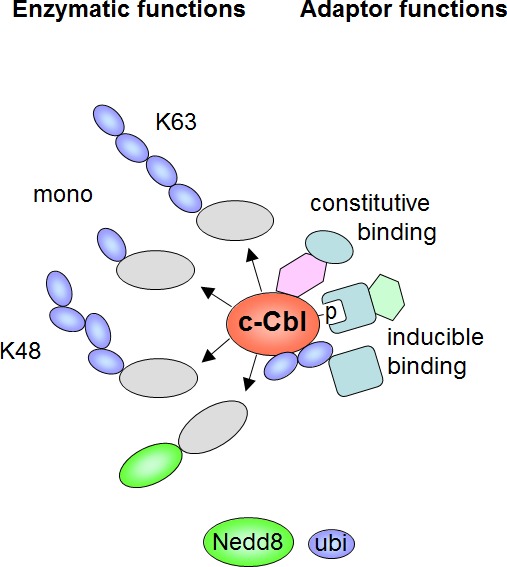
Schematic summary of c-Cbl functions as an adapter protein and as an enzyme Inducible interactions by the ubiquitin binding domain and phosphotyrosine/SH2 interactions are shown. The various enzymatic functions mediating different modifications of the substrate proteins (grey) are displayed.

In addition to these enzymatic activities, the various domains in Cbl proteins enable its function as an adapter by allowing constitutive or phososphorylation-dependent association with a plethora of different proteins [15, 23]. The dual function of c-Cbl as an enzyme and as an adapter protein is schematically displayed in Figure 2. Given the multiplicity of interaction partners and its various enzymatic functions it is not surprising that Cbl proteins affect signaling pathways involved in a wide range of processes, including the immune response, endocytic sorting, apoptosis and autophagy [24-26]. Single nucleotide polymorphism screens and sequencing showed that myeloproliferative neoplasms frequently show mutations in c-Cbl, but not in the other members of the Cbl family [27-29]. While mutations of c-Cbl are frequently discovered in myeloid neoplasms, mutations of the other Cbl family members, Cbl-b and Cbl-c, are hardly detected [30]. So far only five mutations within the RING finger domain of Cbl-b were reported, which likely disrupt its function as a ubiquitin E3 ligase [31, 32].

### c-Cbl mutations in myelodysplastic and myeloproliferative neoplasms

Although approximately 10% of myeloid neoplasms show c-Cbl mutations, their frequency varies considerably between the subtypes. For example, while de novo AML has a relatively low frequency of such mutations at around 1-3%, juvenile myelomonocytic leukemia (JMML) and chronic myelomonocytic leukaemia (CMML) have the highest frequencies of c-Cbl mutations of around 13-15% [33-35]. Mutations in c-Cbl are typically nucleotide substitutions or small insertions/deletions that are heavily enriched in exons 8 and 9 and occur in the linker or RING finger domain of c-Cbl [27, 28, 36, 37]. Approximately 85% of the mutations are point mutations and approximately 15% are small deletions of the linker and RING finger regions. Approximately 10% of patients with MDS/MPN show c-Cbl mutations and frequently display clinical features such as splenomegaly, monocytosis and anemia [38]. Of the point mutations, about 70% are homozygous due to acquired uniparental disomy (aUPD), where both copies of a chromosome pair or parts of chromosomes have originated from one parent. Thus aUPD is a mechanism by which pathogenetic mutations in cancer may be reduced to homozygosity. Thus, an allelic conversion of the 11q arms leads to duplication of the mutated parental copy of 11q and loss of the remaining wild-type allele, resulting in homozygous c-Cbl mutations [36]. These mutations frequently result in the loss of ubiquitin E3 ligase activity [36], thus prohibiting lysosomal or ubiquitin/proteasome-mediated degradation of tyrosine kinases and thereby unleashing tyrosine kinase signaling. Therefore, although these mutations cause biochemical loss-of-function of c-Cbl, their functional consequences resemble classical gain-of-function mutations.

### Mechanisms of c-Cbl mediated oncogenic transformation

The causal contribution of c-Cbl to the onset of myeloid malignancies was revealed in mouse models. c-Cbl-deficient mice are viable but show lymphoid hyperplasia, enhanced T-cell signaling and primary splenic extramedullary hemopoiesis [39]. In contrast, mice lacking Cbl-b do not share phenotypes with c-Cbl-null mice and are characterized by a reduced T-cell activation threshold. Mice with combined deletion of c-Cbl and Cbl-b in hematopoietic stem cells develop an early-onset lethal myeloproliferative disease within the first months after birth [40]. Mice engineered to express a c-Cbl RING domain point mutation (C379A) develop a myeloproliferative disease that progresses to leukemia [41], thus classifying c-Cbl mutations as driver mutations. This phenotype is even more severe than that of c-Cbl-deficient mice, suggesting that c-Cbl mutations act in a dominant negative manner and also affect the other endogenous members of the Cbl family. In addition to its function as a dominant negative protein mutant c-Cbl may also act as a positive signaling protein via its adaptor function. While a single c-Cbl point mutation can cause myeloid malignancies in mice, the situation is markedly different in humans. This difference is unlikely to be attributable to differences in expression levels or other experimental settings, as in humans over 10% of the tumors carry c-Cbl mutations together with 2-4 additional characteristic mutations [11, 37]. Furthermore, mutations at amino-acid residue C381 in human c-Cbl (equivalent to amino-acid residue C379 in mouse c-Cbl) amounts to ~0.8% of the mutations identified. The relative contribution of c-Cbl to development of MDS/MPN might be underestimated because some other genes known to be mutated or amplified in MDS/MPN, such as the duplicated gene encoding the SH3KBP1 (SH3-domain kinase binding protein) adapter protein, are part of the c-Cbl-regulated signaling network and may have the same effect as a c-Cbl mutation [42]. In addition, inactivation of c-Cbl may be mediated by events that do not affect the c-Cbl sequence. Phosphatases may keep c-Cbl in an inactivated state and increased auto-ubiquitination can limit the available protein amounts of the E3 ligase in tumor cells. Novel proteogenomic approaches for characterization of tumor cells will enable completely new insights in the relative protein abundance of c-Cbl and its client proteins [43]. In humans, c-Cbl mutations are not typical founding driver mutations allowing the first step in the leukemogenic process, but rather occur as subclonal secondary driver mutations, ensuring the further progress of the disease. Support for this model comes from the analysis of a patient with essential thrombocythemia, who had an intact c-Cbl gene at the early stage of disease, but acquired a c-Cbl R420Q mutation during disease progression to myelofibrosis [29]. The concept of c-Cbl mutations as secondary driver mutations is recapitulated in an animal model where transgenic mice expressing the oncogenic fusion protein NHD13 (NUP98-HOXD13) develop a myelodysplastic syndrome, which proceeds to acute leukemia. These animals pick up several secondary driver mutations in several genes including c-Cbl [44]. A deep sequencing study performed with a cell colony derived from a single cell detected ten mutations, compatible with the concept of a linear evolution of a dominant clone [34]. Recent advances in single cell genomics will now enable a detailed view into the stepwise acquisition of primary and secondary driver mutations [45]. It was suggested that c-Cbl mutations might indicate a poor clinical prognosis [38], but this is not consistently seen in other studies [46, 47]. It will be therefore important to consider also the prognostic value of these co-occurring secondary mutations in the future.

The importance of c-Cbl to myeloid differentiation and cancer is likely due to its involvement in numerous signaling pathways and biological functions. Mutation of c-Cbl can lead to aberrant activation of the JAK-STAT and PI3K-AKT pathways to transmit mitogenic and/or survival signals [48-50]. The mutant c-Cbl protein can also affect cytoskeleton organization via activation of Rac1 or Cdc42, and R-RAS [51] and affects integrin-mediated cell adhesion, spreading, and migration [52, 53]. Cbl proteins are important for the control of signaling thresholds in immune cells, and point mutations of c-Cbl consistently lead to enhanced granulocyte-macrophage colony-stimulating factor (GM-CSF)-triggered signaling via STAT5 and JAK2 [54, 55].

Cbl also contributes to myeloid differentiation by regulating the activity of colony stimulating factor-1 (CSF-1) receptor (CSF-1R). In the hematopoietic system, CSF-1 is believed to act specifically on myeloid progenitors, starting from the CMP stage, and to favour the differentiation of CMPs into the monocyte/macrophage lineage [56]. Indeed, transgenic mice engineered to secrete human CSF-1 show augmented frequencies and functions of human myeloid cells [57]. CSF-1 can directly induce the myeloid master regulator PU.1 and thus instruct myeloid cell-fate change in mouse HSCs both *in vitro* and *in vivo* [58]. Furthermore, intra-hepatic transfer of human fetal liver derived hematopoietic stem and progenitor cells (CD34^+^) in humanized CSF-1 newborn mice resulted in more efficient differentiation and enhanced frequencies of human monocytes/macrophages in the bone marrow, spleens, peripheral blood, lungs, liver and peritoneal cavity, pointing to its potential role in myeloid cell fate [57]. The magnitude and duration of signaling through activated CSF-1R is tightly regulated by its c-Cbl mediated ubiquitination-dependent down-regulation [59-61]. These c-Cbl-mediated effects on soluble factors such as CSF-1 raise the intriguing possibility that an oncogenic mutation in one tumor cell might affect neighbouring wildtype cells by paracrine mechanisms.

### Future directions

Accumulating clinical evidence shows that progression mutations in c-Cbl and other key regulators occur during further clonal development of myeloid malignancies. Unlike the classical gain-of-function mutations exemplified by constitutive active JAK2 (V617F) or BCR-ABL [62], mutant c-Cbl has lost its enzymatic activity which renders it not an obvious drug target. This raises the need to identify druggable downstream components of the c-Cbl signaling pathways. Therefore, the downstream effectors of c-Cbl such as the JAK/STAT, PI3K, and ERK signaling pathways have been suggested as potential therapeutic targets. However, drugs acting on these signaling endpoints will not be specific for myeloid malignancies. As mutant c-Cbl proteins could display residual enzymatic activities as E3 ligases it may also be feasible to inhibit deubiquitinating enzymes that counteract c-Cbl. In order to develop drugs that are specifically tailored for the treatment of myeloid tumors with c-Cbl mutations we need a better understanding of the functional consequences of these mutations. The importance of this concept has been demonstrated by the use of mice with a c-Cbl RING finger mutation that develop a myeloproliferative disease progressing to leukemia. These mice exhibit augmented FLT3 (fms-related tyrosine kinase 3) signaling and inhibition of FLT3 kinase activity by quizartinib (AC220) effectively suppresses MPD development [41]. Deciphering how individual c-Cbl mutations affect its different enzymatic functions (neddylation, monoubiquitination, regulatory or proteolytic polyubiquitination) will provide therapeutic clues. As the activity of c-Cbl proteins is regulated by conformational changes [17, 18], it will be important to determine changes in the interactomes between wildtype and oncogenically mutated proteins. Also the intracellular localization of mutant c-Cbl and its posttranslational modifications need to be investigated, as phosphorylation of Y700 enables the interaction with further signal transmitting enzymes such as Vav1 [63], while phosphorylation at Y731 and Y774 allows binding of the p85 subunit of PI3K and the Crk-family of adapter proteins, respectively [48, 64]. Along this line, a comparative analysis of phosphoproteomes in cells expressing wildtype or mutant c-Cbl would help in the exploration of deregulated signaling pathways. New genetic tools such as inducibly expressed shRNAs or CRISPR-Cas9-mediated genome editing will enable synthetic lethality screens to identify druggable interactions between mutant c-Cbl and further components of the signaling network.

## Abbreviations

AML: acute myelogenous leukemia
aUPD: acquired uniparental disomy
c-Cbl: Casitas B-lineage Lymphoma
CML: chronic myeloid leukemia
CMML: chronic myelomonocytic leukaemia
CMP: common myeloid progenitor
CSF-1R: colony stimulating factor-1 (CSF-1) receptor
FLT3: fms-related tyrosine kinase 3
GM-CSF: granulocyte-macrophage colony-stimulating factor
HSC: hematopoietic stem cell
JAK2: Janus kinase 2
JMML: juvenile myelomonocytic leukemia
MDS: myelodypastic syndromes
MDS/MPN: myelodysplastic/myeloproliferative neoplasms
MPDs: myeloproliferative disorders
MPPs: multipotent progenitors
NEDD8: neural precursor cell expressed, developmentally down-regulated 8
NHD13: NUP98-HOXD13
RING: really interesting new gene
SH2: Src homology 2
SH3: Src homology 3
SH3KBP1: SH3-domain kinase binding protein
TKB: tyrosine-kinase-binding
